# Arthroscopic-assisted uni-portal spinal surgery versus unilateral biportal endoscopy for lumbar degenerative diseases: a systematic review and meta-analysis of clinical outcomes and complications

**DOI:** 10.1186/s12891-026-10328-8

**Published:** 2026-08-08

**Authors:** Jianbo Yan, Lixin Deng, Donger Zeng, Bingyin Sun

**Affiliations:** Shunde Hospital of Guangzhou University of Chinese Medicine, No. 12 Jinsha Avenue, Shunfengshan, Daliang Subdistrict, Shunde District, Foshan City, Guangdong Province People’s Republic of China

**Keywords:** Arthroscopic-assisted uni-portal spinal surgery, Unilateral biportal endoscopy, Lumbar degenerative disease, Meta-analysis

## Abstract

**Background:**

The optimal endoscopic surgical approach for lumbar degenerative diseases remains debated. This meta-analysis compared the clinical performance and safety of arthroscopic-assisted uni-portal spinal surgery (AUSS) versus unilateral biportal endoscopy (UBE/BESS). To provide guidance on the choice of surgical approaches of the lumbar degenerative diseases.

**Methods:**

A systematic literature search was performed across PubMed, Embase, Web of Science, Cochrane Database, China National Knowledge Network, Wanfang Database, and China Biomedical Literature Database up to March 28, 2026. Studies comparing AUSS and UBE/BESS for lumbar degenerative conditions were included. We analyzed the extracted data with Review Manager 5.4.

**Results:**

Three studies encompassing 810 patients (419 AUSS, 391 UBE/BESS) were analyzed. The AUSS group demonstrated significantly reduced operative time (MD = -14.72, 95% CI: −22.32 to − 7.12, *P* = 0.0001), lower intraoperative blood loss (MD =−10.59, 95% CI: −16.92 to − 4.26, *P* = 0.001), shorter incision length (MD = − 1.43, 95% CI: −1.64 to − 1.22, *P* < 0.00001), lower Visual Analog Scale (VAS) scores for back pain 3 months post-surgery (MD = -0.14, 95%CI: -0.24 to -0.03, *P* = 0.01), The excellent and good outcome rate was also higher in the AUSS group (OR = 1.52, 95%CI: 1.01 to 2.28, *P* = 0.04); No significant between-group differences emerged for hospital stay, Oswestry Disability Index (ODI) at any postoperative timepoint, or complication rates.

**Conclusions:**

Both techniques are safe and effective for lumbar degenerative diseases, with comparable functional recovery and safety profiles. AUSS appears to be associated with improved surgical efficiency, but these findings are exploratory and require confirmation by high-quality RCTs.

## Introduction

Lumbar degenerative diseases (LDD), one of the most common conditions in spinal surgery, primarily include lumbar disc herniation (LDH), lumbar spinal stenosis (LSS), and lumbar spondylolisthesis. With the acceleration of population aging, the incidence of LDD has been rising year by year, severely impacting patients’ quality of life and imposing a heavy socioeconomic burden [1]. For patients unresponsive to conservative treatment, surgical intervention is an effective means of alleviating symptoms and improving function [2]. Although traditional open surgery is effective, it has drawbacks such as significant trauma, excessive bleeding, and slow recovery [3]. In recent years, with the widespread adoption of minimally invasive principles and advances in endoscopic technology, endoscopic spinal surgery has become an important treatment option for LDD due to its many advantages, including shorter procedure times, minimal trauma, and reduced intraoperative bleeding [4].The unilateral biportal endoscopy (UBE/BESS) technique was systematically refined and promoted by South Korean scholars in the 2010s [5]. This technique employs a dual-channel design with separate observation and working channels and utilizes standard spinal surgical instruments. It offers advantages such as a clear field of view, flexible maneuverability, and thorough decompression. Currently, UBE/BESS is widely used in the treatment of various lower back disorders, including lumbar spinal stenosis and lumbar disc herniation, and numerous studies have confirmed its safety and efficacy [6].Arthroscopic-assisted Uni-portal Spinal Surgery (AUSS) is a novel single-portal endoscopic technique first proposed by Chinese scholar Professor Song En in 2021 [7]. This technique combines the triangular operating concept of arthroscopy with the minimally invasive advantages of single-portal endoscopy, enabling both visualization and manipulation through a single incision. It retains the low-trauma characteristics of single-portal endoscopy while overcoming the limitations of traditional single-portal endoscopy regarding restricted operating space. AUSS employs an open field of view, allowing the surgeon to operate from a perspective closer to that of open surgery. Theoretically, this offers the advantages of a gentler learning curve and simpler manipulation [8]. Since the introduction of the AUSS technique, several single-center studies have reported its clinical efficacy in treating LDD [9]; however, no meta-analysis has yet directly compared the efficacy of AUSS with that of UBE/BESS. Although meta-analyses comparing one-hole split endoscopy (OSE) with UBE/BESS have been published, AUSS [10], as a distinct new technique, differs from OSE in its underlying technical principles. Therefore, this study aims to systematically collect and analyze all clinical studies comparing AUSS and UBE/BESS for the treatment of lumbar degenerative diseases, evaluate the clinical efficacy of the two surgical approaches, and provide evidence-based medical evidence to guide the selection of surgical methods in clinical practice.

## Materials and methods

### Study selection and search strategy

This study conducted a systematic review and meta-analysis in accordance with the Preferred Reporting Items for Systematic Reviews and Meta-Analyses (PRISMA) guidelines. The study protocol has been registered on the international systematic review registry PROSPERO under registration number CRD420261336136. Since the AUSS technique was first proposed in 2021, the number of relevant publications is limited, and there is a lack of clearly defined subject terms. To enhance the comprehensiveness of this review, we employed the following search terms in the abstract: (((arthroscopic-assisted uni-Portal spinal surgery*) OR (arthroscopic-assisted uniportal spinal surgery*) OR (AUSS)) AND (lumbar)). The databases searched included PubMed, Embase, Web of Science, the Cochrane Database, China National Knowledge Infrastructure (CNKI), Wanfang Database, and the China Biomedical Literature Database (CBM), with the search period ending on March 28, 2026.

### Inclusion and exclusion criteria

The inclusion criteria for this meta-analysis were established according to the PICOS principles: (1) Participants: Patients clinically diagnosed with degenerative lumbar spine disease, including lumbar disc herniation, lumbar spinal stenosis, and lumbar spondylolisthesis. There were no restrictions on age, gender, ethnicity, or disease duration (2). Intervention: Treatment using AUSS (3). Comparison: Treatment using UBE/BESS (4). Outcomes: At least one of the following outcome measures must be reported: ① Surgical-related measures: operative time, intraoperative blood loss, incision length, length of hospital stay; ② Pain scores: Visual Analog Scale (VAS) assessment of low back pain and/or leg pain; ③ Functional scores: Oswestry Disability Index (ODI); ④ Clinical excellent and good rates; (5) Study design: randomized controlled trials (RCTs), prospective cohort studies, retrospective cohort studies, and case-control studies.Studies will be excluded if they meet any of the following criteria: (1) Studies lacking relevant data, including but not limited to case series (sample size < 10 cases), case reports, reviews, commentaries, conference abstracts, technical descriptions, animal studies, and cadaver studies; (2) Single-arm studies without a control group; (3) Literature with duplicate publication of data or incomplete statistical data; (4) Studies with data falling outside clinically plausible ranges or with incomplete reporting of essential methodological details (5). Studies not published in Chinese or English. The researchers first conducted a thorough screening of the titles and abstracts of all literature, then evaluated the full texts of eligible studies based on the established inclusion and exclusion criteria. Finally, a comprehensive review of the included studies was performed, and relevant data were systematically extracted.

### Assessment of literature quality

Two reviewers independently conducted the literature screening and quality assessment. High-quality studies were selected based on the results of the quality assessment. Any discrepancies between the two reviewers were resolved through discussion with a third arbitrator to reach a consensus. The quality of the included studies was assessed using the Newcastle-Ottawa Scale (NOS) for observational studies and the Cochrane Risk of Bias (RoB 2) tool for randomized trials.

### Data extraction

Two researchers independently extracted the data, cross-checked it upon completion, and then a third researcher compiled and verified the data. The demographic data extracted included the study authors, year of publication, country of origin, study design, number of patients, disease type, age, gender, BMI, and follow-up duration; surgery-related outcome measures included operative time, intraoperative blood loss, incision length, and length of hospital stay; clinical outcome measures included VAS scores for low back pain and leg pain at various postoperative time points, ODI scores at multiple time intervals, the rate of excellent and good treatment outcomes, complication rates, CK, CRP, EQ-5D-5 L, number of X-ray exposures, small joint preservation rates, and CT imaging parameters. If study data were incomplete, attempts were made to contact the corresponding author to obtain raw data.

This meta-analysis was conducted using RevMan 5.4 software. Continuous variables were reported as mean differences (MD) with 95% confidence intervals (CI), while dichotomous variables were reported as odds ratios (OR) with 95% CI. Heterogeneity was assessed first. If *P* ≤ 0.10 or I² ≥ 50%, significant heterogeneity was deemed present, and a random effects model was used; otherwise, a fixed effects model was applied. Sensitivity analysis was performed using a stepwise exclusion of individual studies. Due to the limited number of included studies, no publication bias assessment was conducted.

## Results

### Literature search results

**Fig. 1:**
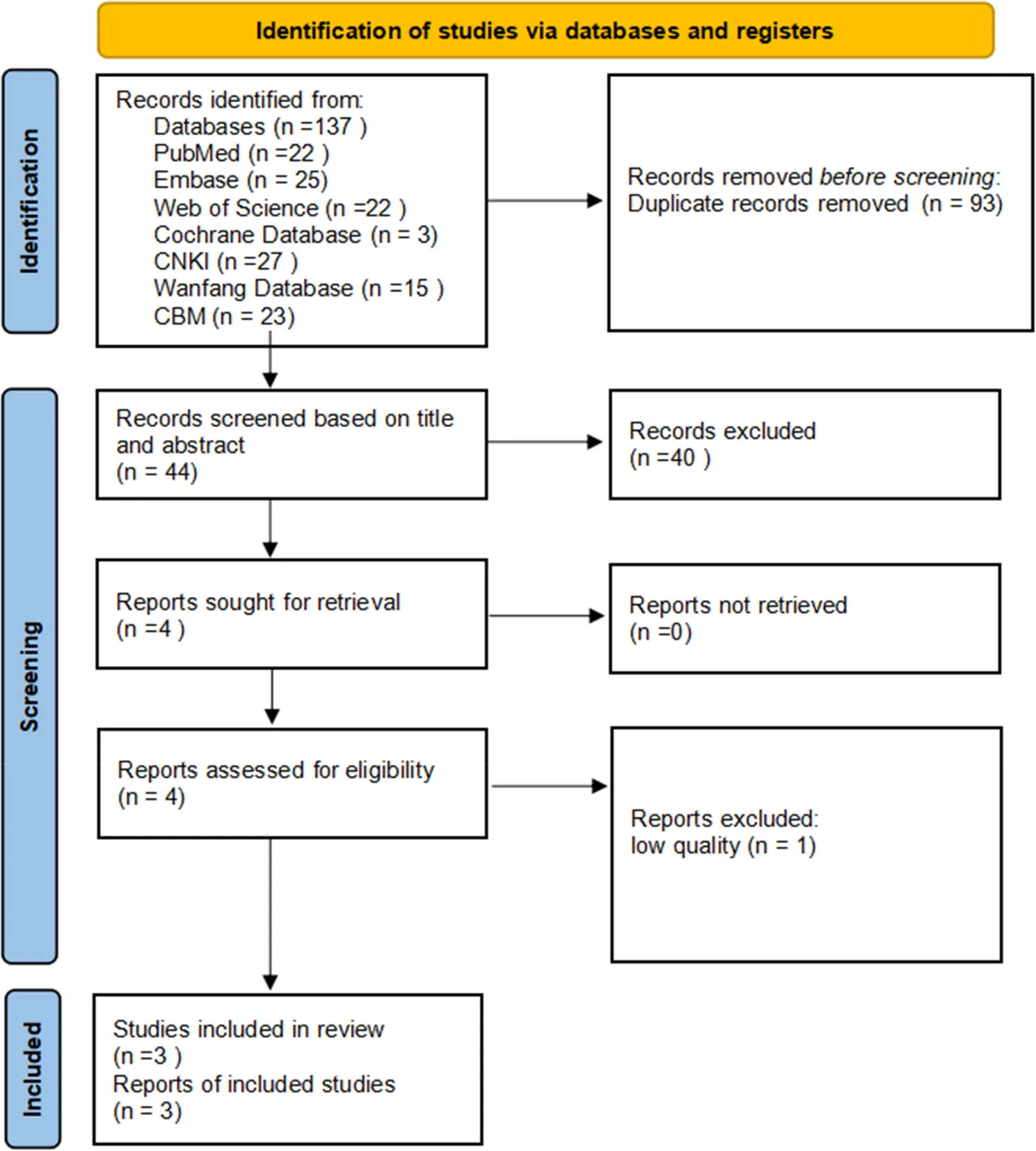
PRISMA flow chart of literature search

The initial literature search for this study yielded a total of 137 articles, including 22 from PubMed, 24 from Embase, 3 from the Cochrane Library, 22 from Web of Science, 27 from CNKI, 15 from Wanfang, and 23 from CBM. After removing duplicates using EndNote, 44 articles remained. Following a review of the titles and abstracts, 40 articles were excluded for reasons including: non-comparative studies, mismatched interventions, single-arm studies, and case reports, leaving 4 articles. After reviewing the full texts, one study was excluded due to data anomalies: the incision length exceeded the clinical range and the standard deviation was implausibly small for the reported mean value, raising doubts about data authenticity and potentially compromising the reliability of the conclusions [11]. Ultimately, three studies were included in the meta-analysis, comprising two English-language studies [9, 12] and one Chinese-language study [13]. The literature screening process and results are detailed in the PRISMA flowchart (Fig. 1).

### Study characteristics and quality assessment

The characteristics and details of the included studies are shown in (Table 1). The three included studies were published between 2024 and 2025; all were retrospective cohort studies and were conducted in China. A total of 810 patients were included: 419 in the AUSS group and 391 in the UBE/BESS group. All three studies met the threshold for low risk of bias (≥ 5 points), and the overall quality of the included studies was rated as high according to the NOS. (Table 2) provides a summary of the quality assessments based on the NOS for non-randomized studies.

**Table 1:** Characteristics of included studies

Study	Country	Surgery	Sample	Disease	Age	Gender (M/F)	BMI	Follow-up duration
	Design	Procedures	Size					
Wang 2024 [9]	China	AUSS	240	LSS	56.7±7.8	145/95	24.3±2.3	Preoperative/3m/6m
	Retrospective	UBE	235		57.9±6.9	123/112	24.6±2.1	Preoperative/3m/6m
Yang 2025 [13]	China	AUSS	50	LDH	50.2±16.5	30/20	23.4±2.8	Preoperative/3m
	Retrospective	UBE	70		51.4±14.6	41/29	23.5±2.8	Preoperative/3m
Zhou 2025 [12]	China	AUSS	129	LSS	51.07±18.50	63/66	23.85±2.76	Preoperative/3d/3m/6m
	Prospective case-control	UBE	86		49.66±18.06	42/44	23.87±3.13	Preoperative/3d/3m/6m

**Table 2:** Newcastle-Ottawa scale for assessing the quality of studies in meta-analysis

Study	Selection of the study population	Comparability of groups	Outcome measures	Quality assessment
Wang 2024 [9]	4	2	2	8
Yang 2025 [13]	4	1	1	6
Zhou 2025 [12]	4	2	3	9

### Meta-analysis results

#### Operative duration

**Fig. 2:**
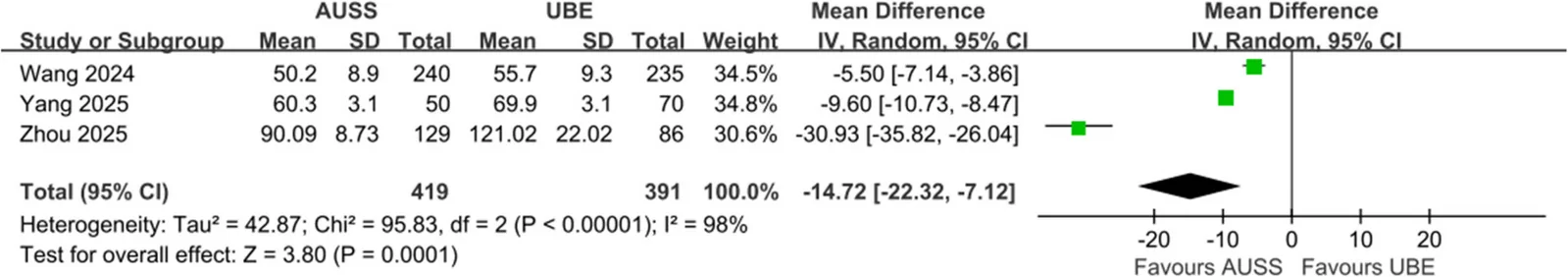
Forest plot of operative durations

To evaluate operative duration, we pooled data from all three included studies, totaling 810 patients (419 AUSS, 391 UBE/BESS). Owing to substantial across-study heterogeneity (I² = 98%, *P* < 0.00001), with I² exceeded 50%, a random-effects model was applied. The summary estimate revealed a significantly shorter operative time favoring AUSS over UBE/BESS (MD = − 14.72, 95% CI: −22.32 to − 7.12, *P* = 0.0001; Fig. 2). A sensitivity analysis of the outcome measures was conducted using the “exclude one study at a time” method. Excluding any single study did not alter the direction of the pooled effect size, indicating that no single study had a dominant influence on the overall results, and that the findings of this study are stable. Potential sources of heterogeneity may be related to differences in the operational proficiency of different surgeons.

#### Intraoperative blood loss

**Fig. 3:**
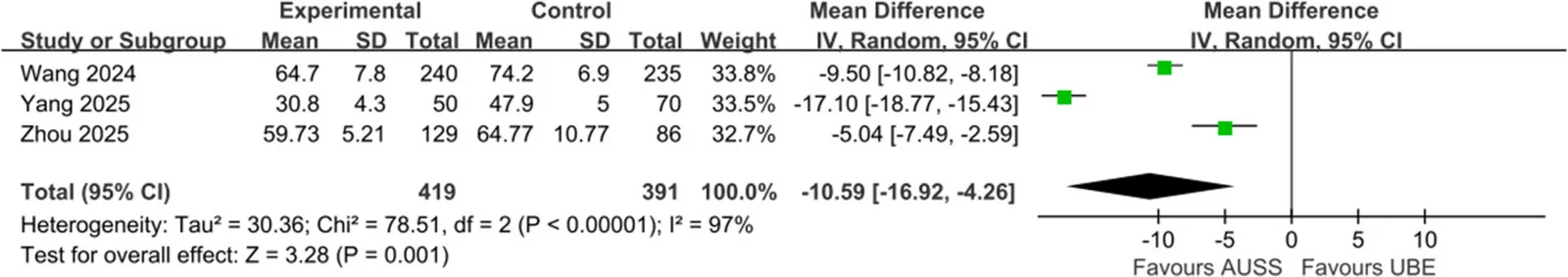
Forest plot of intraoperative blood loss

To evaluate intraoperative blood loss, we pooled data from all three included studies, totaling 810 patients (419 AUSS, 391 UBE/BESS). Owing to substantial across-study heterogeneity (I² = 97%, *P* < 0.00001), with I² exceeded 50%, a random-effects model was adopted. The summary estimate revealed a significantly lower intraoperative blood loss favoring AUSS over UBE/BESS (MD = − 10.59, 95% CI: −16.92 to − 4.26, *P* = 0.001; Fig. 3). A sensitivity analysis of the outcome measures was conducted using the one-by-one exclusion method. Excluding any single study did not alter the direction of the pooled effect size, indicating that no single study exerted a dominant influence on the overall results, and that the findings of this study are stable. Potential sources of heterogeneity may be related to differences in surgical techniques among different surgeons.

#### Incision length

**Fig. 4:**
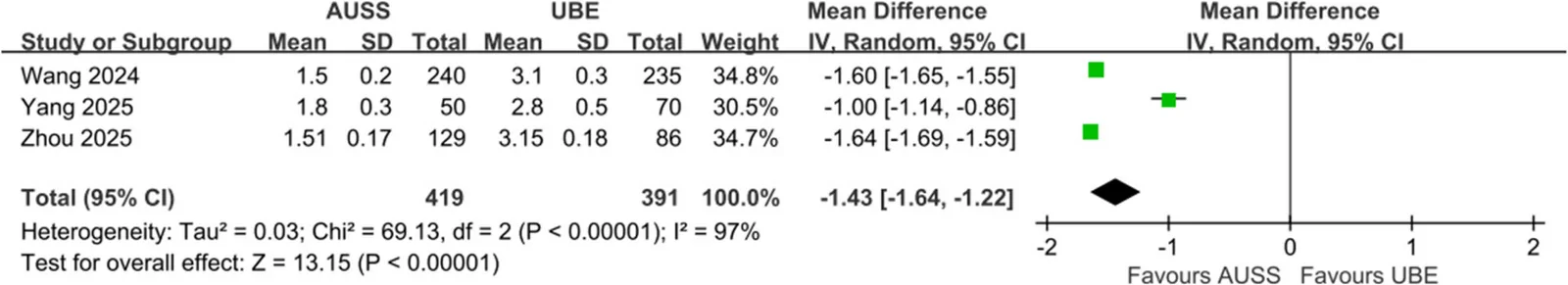
Forest plot of incision length

To evaluate incision length, we pooled data from all three included studies, totaling 810 patients (419 AUSS, 391 UBE/BESS). Owing to substantial across-study heterogeneity (I² = 97%, *P* < 0.00001), with I² exceeded 50%, a random-effects model was adopted. The summary estimate revealed a significantly shorter incision length favoring AUSS over UBE/BESS (MD = − 1.43, 95% CI: −1.64 to − 1.22, *P* < 0.00001; Fig. 4). A sensitivity analysis of this outcome measure was conducted using the stepwise exclusion of individual studies. After excluding Yang’s study, the I² statistic decreased significantly from 97% to 28%, and the pooled (MD = − 1.62, 95% CI: −1.66 to − 1.58, *P* < 0.00001), consistent with the direction of the main analysis. After excluding Wang 2024 or Zhou 2025, heterogeneity remained high, and the direction of the pooled effect did not change. The results indicate that Yang’s study is the primary source of heterogeneity regarding incision length; however, excluding this study did not alter the direction of the conclusions, demonstrating stable findings. The source of heterogeneity may stem from differences in disease type, as Yang’s study focused on LDH, whereas Wang and Zhou focused on LSS.

#### Rates of excellent and good outcomes

**Fig. 5:**
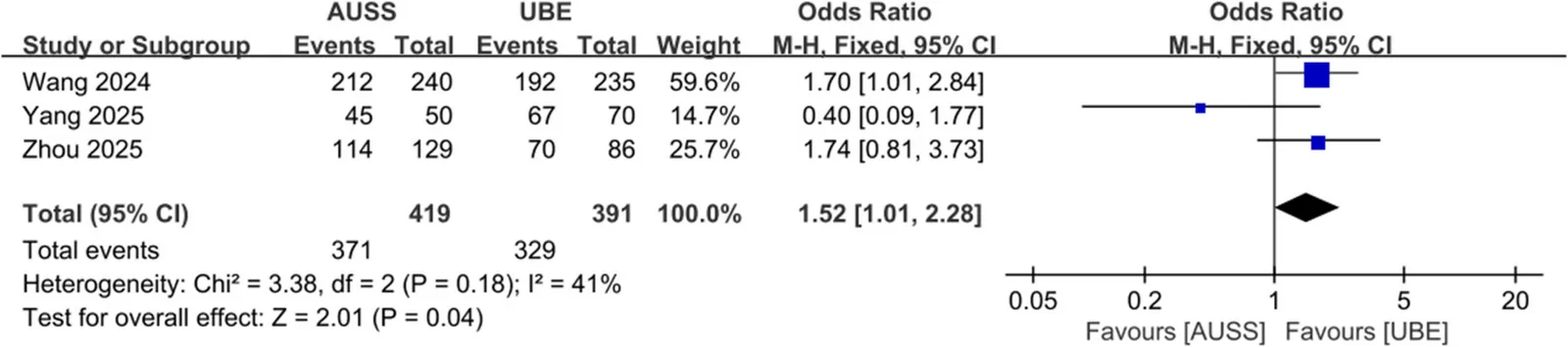
Forest plot of length of rates of excellent and good outcomes

To evaluate rates of excellent and good outcomes, we pooled data from all three included studies, totaling 810 patients (419 AUSS, 391 UBE/BESS). The analysis revealed no significant heterogeneity among the studies (I² = 41%, *P* = 0.18), with I² did not exceed 50%, a fixed-effects model was applied. The summary estimate revealed a significantly higher rates of excellent and good outcomes favoring AUSS over UBE/BESS (OR = 1.52, 95% CI: 1.01 to 2.28, *P* = 0.04; Fig. 5).

#### Hospital stay

**Fig. 6:**
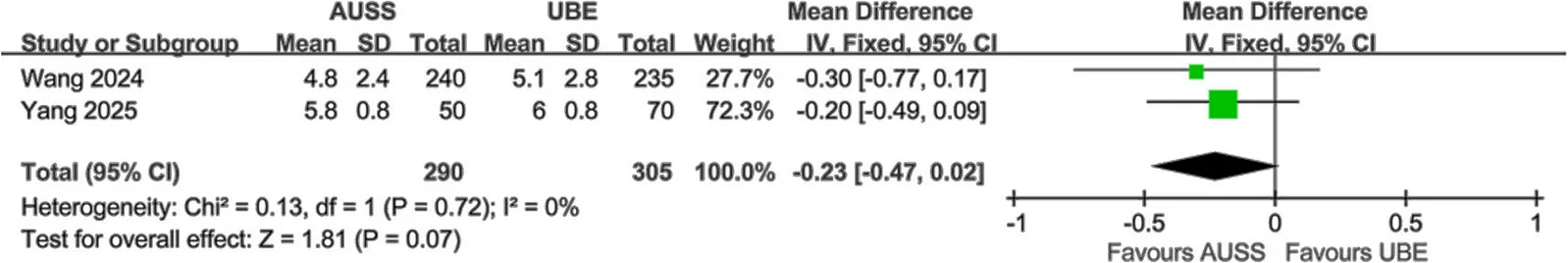
Forest plot of length of hospital stay

To evaluate length of hospital stay, we pooled data from all two included studies, totaling 595 patients (290 AUSS, 305 UBE/BESS). The analysis revealed no heterogeneity among the studies (I² = 0%, *P* = 0.72), with I² did not exceed 50%, a fixed-effects model was employed. The meta-analysis indicated that the difference between the two groups was not statistically significant (MD = − 0.23, 95% CI: −0.47 to 0.02, *P* = 0.07; Fig. 6).

#### VAS scores for low back pain within postoperative 3 months

**Fig. 7:**
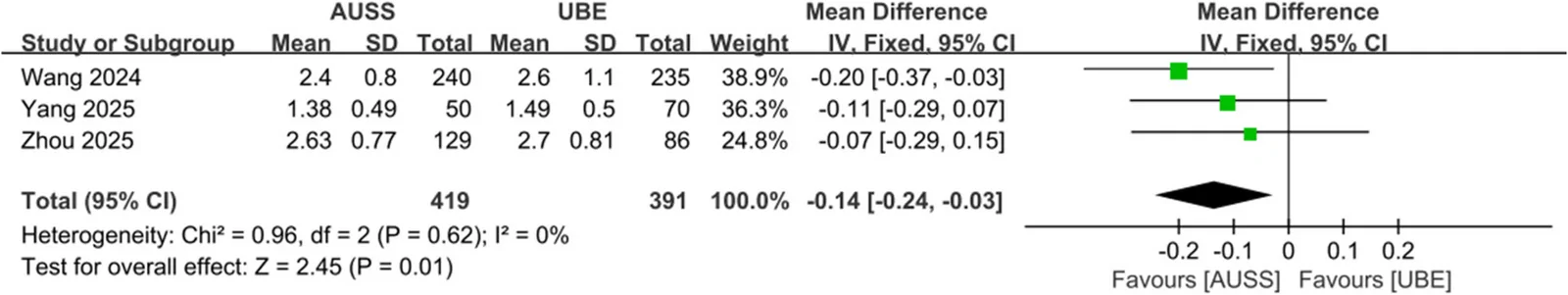
Forest plot of VAS scores for low back pain at postoperative 3 months

To evaluate VAS scores for low back pain within postoperative 3 months, we pooled data from all three included studies, totaling 810 patients (419 AUSS, 391 UBE/BESS). The analysis revealed no heterogeneity among the studies (I² = 0%, *P* = 0.62), with I² did not exceed 50%, a fixed-effects model was applied. The summary estimate revealed a significantly lower the 3-month postoperative VAS score for low back pain favoring AUSS over UBE/BESS (MD = − 0.14, 95% CI: −0.24 to − 0.03, *P* = 0.01; Fig. 7).

#### VAS scores for leg pain within postoperative 3 months

**Fig. 8:**
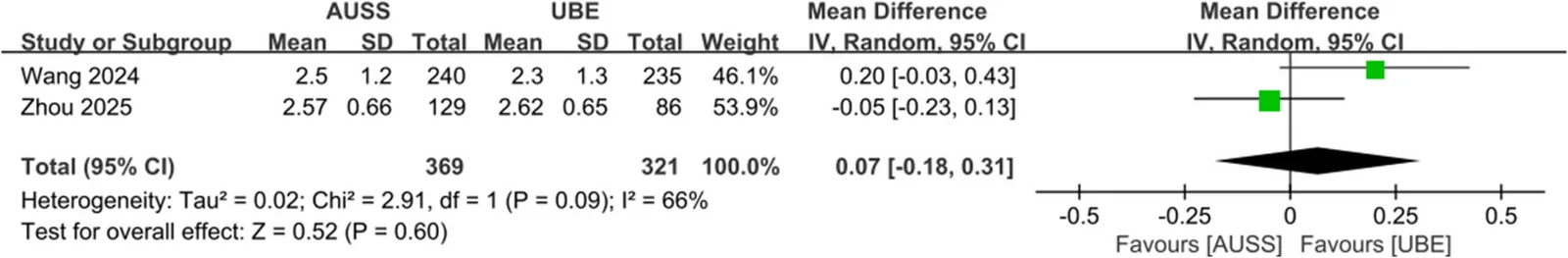
Forest plot of VAS scores for leg pain at postoperative 3 months

To evaluate VAS scores for leg pain within postoperative 3 months, we pooled data from all two included studies, totaling 690 patients (369 AUSS, 321 UBE/BESS). Owing to the analysis revealed moderate heterogeneity among the studies (I² = 66%, *P* = 0.09), with I² exceeded 50%, a random-effects model was adopted. The meta-analysis indicated that the difference between the two groups was not statistically significant (MD = 0.07, 95% CI: -0.18 to 0.31, *P* = 0.60; Fig. 8).

#### VAS scores for low back pain within postoperative 6 months

**Fig. 9:**
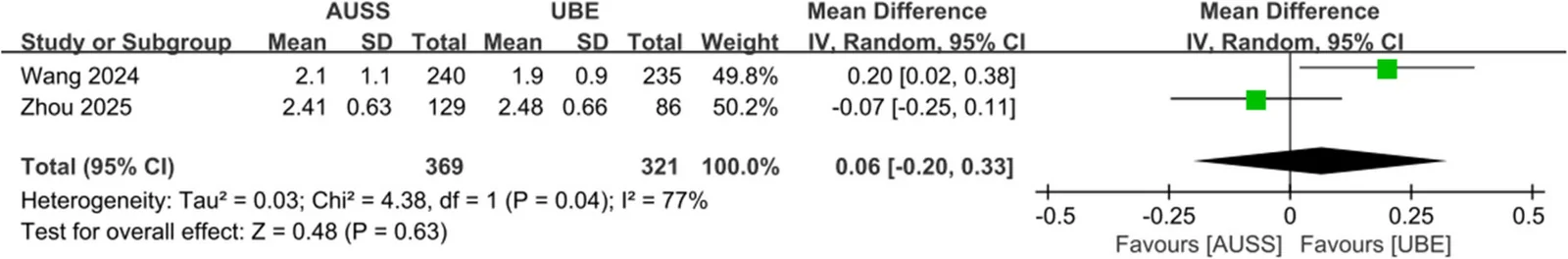
Forest plot of VAS scores for low back pain at postoperative 6 months

To evaluate VAS scores for low back pain within postoperative 6 months, we pooled data from all two included studies, totaling 690 patients (369 AUSS, 321 UBE/BESS). Owing to the analysis revealed moderate heterogeneity among the studies (I² = 77%, *P* = 0.04), with I² exceeded 50%, a random-effects model was adopted. The meta-analysis indicated that the difference between the two groups was not statistically significant (MD = 0.06, 95% CI: -0.20 to 0.33, *P* = 0.63; Fig. 9).

#### VAS scores for leg pain within postoperative 6 months

**Fig. 10:**
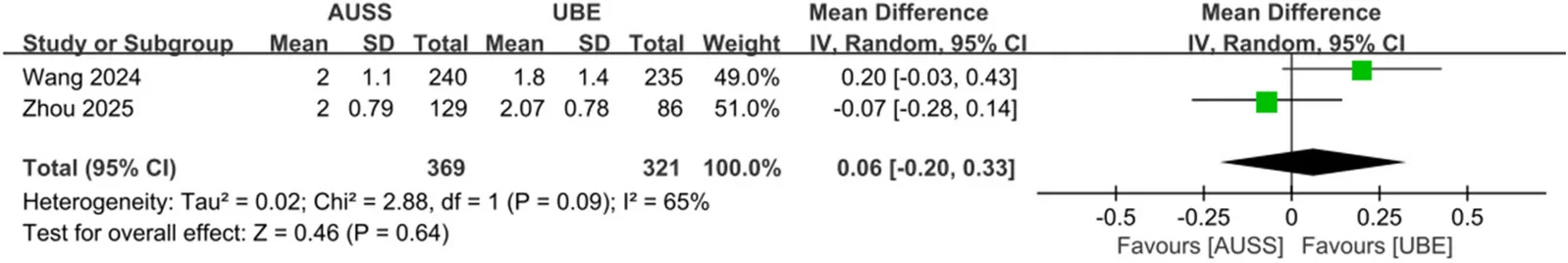
Forest plot of VAS scores for leg pain at postoperative 6 months

To evaluate VAS scores for low leg within postoperative 6 months, we pooled data from all two included studies, totaling 690 patients (369 AUSS, 321 UBE/BESS). Due to the analysis revealed moderate heterogeneity among the studies (I² = 65%, *P* = 0.09), with I² exceeded 50%, a random-effects model was applied. The results indicated that there was no statistically significant difference between the two groups (MD = 0.06, 95% CI: -0.20 to 0.33, *P* = 0.64; Fig. 10).

#### ODI values at postoperative 3 months

**Fig. 11:**
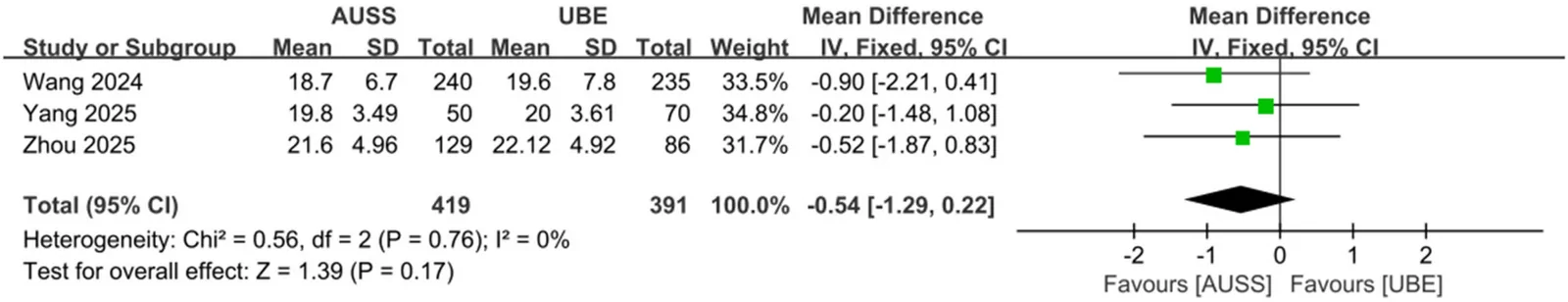
Forest plot of ODI values at postoperative 3 months

To evaluate ODI values at postoperative 3 months, we pooled data from all three included studies, totaling 810 patients (419 AUSS, 391 UBE/BESS). The analysis revealed no heterogeneity among the studies (I² = 0%, *P* = 0.76), with I² did not exceed 50%, a fixed-effects model was used. The meta-analysis indicated that the difference between the two groups was not statistically significant (MD = − 0.54, 95% CI: −1.29 to 0.22, *P* = 0.17; Fig. 11).

#### ODI values at postoperative 6 months

**Fig. 12:**
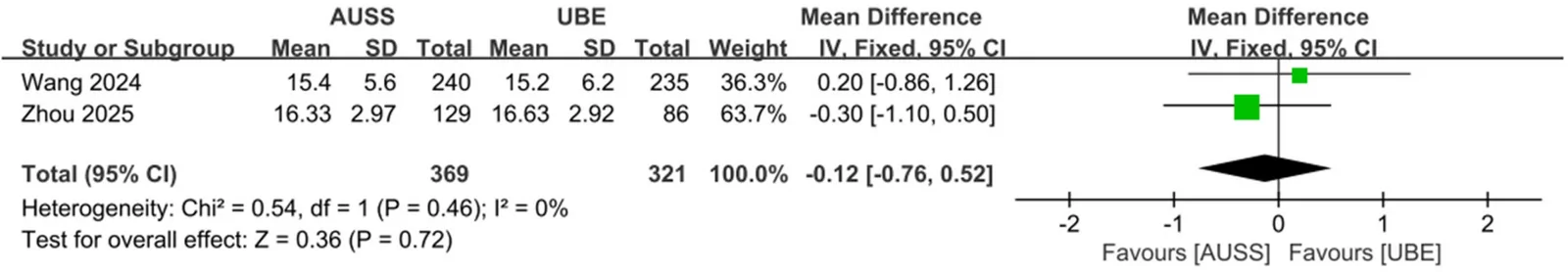
Forest plot of ODI values at postoperative 6 months

To evaluate ODI values at postoperative 6 months, we pooled data from all two included studies, totaling 690 patients (369 AUSS, 321 UBE/BESS). The analysis revealed no heterogeneity between studies (I² = 0%, *P* = 0.46), with I² did not exceed 50%, a fixed-effects model was applied. The meta-analysis indicated that the difference between the two groups was not statistically significant (MD = -0.12, 95% CI: -0.76 to 0.52, *P* = 0.72; Fig. 12).

### Complications

**Fig. 13:**
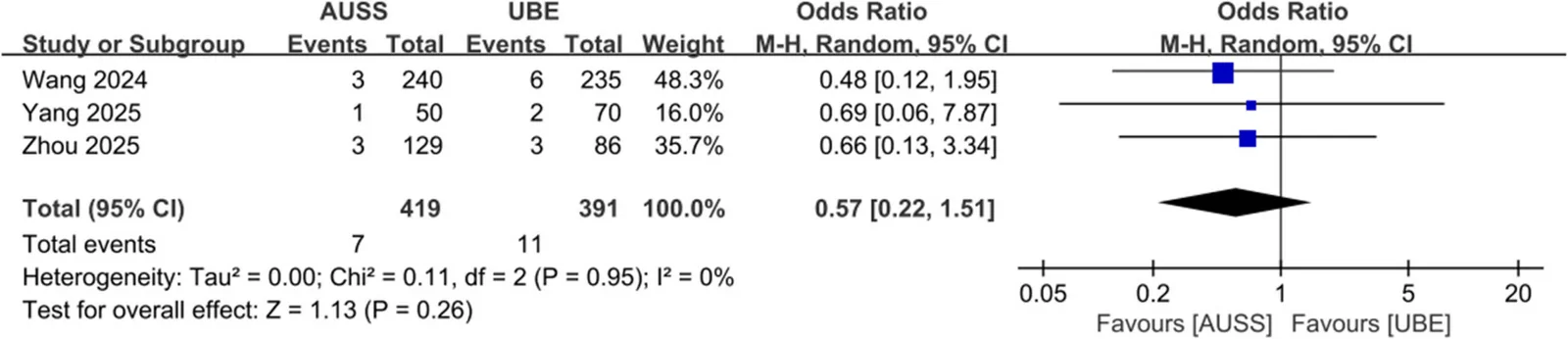
Forest plot of Complications

To evaluate incidence of complications, we pooled data from all three included studies, totaling 810 patients (419 AUSS, 391 UBE/BESS). The analysis revealed no heterogeneity among the studies (I² = 0%, *P* = 0.95), with I² did not exceed 50%, a fixed-effects model was employed. The meta-analysis indicated that there was no significant difference in the overall incidence of complications between the two groups (OR = 0.57, 95% CI: 0.22–1.50, *P* = 0.25; Fig. 13).

### Publication bias

According to the Cochrane Handbook, when the number of included studies is fewer than 10, the symmetry of the funnel plot is unreliable, and Egger’s test has very low sensitivity and a high false-negative rate, making it easy to erroneously conclude that there is “no publication bias.” Therefore, funnel plot analysis and Egger’s test were not performed.

## Discussion

LDD is a spectrum of conditions characterized primarily by disc degeneration, facet joint hyperplasia, and thickening of the ligamentum flavum [14]. The pathological essence of LDD lies in the degeneration and destabilization of the spinal functional unit, leading to nerve compression, pain, and functional impairment. With the accelerating aging of the population, the incidence of LDD continues to rise, making it the most common condition in spinal surgery. The goals of surgical treatment for LDD include adequate decompression of neural structures and the maintenance or restoration of spinal stability. To minimize surgical trauma and promote rapid recovery, surgical techniques are evolving toward minimally invasive approaches [15]. While traditional open surgery can achieve adequate decompression, it has drawbacks such as extensive paraspinal muscle dissection, significant blood loss, persistent postoperative low back pain, and accelerated degeneration of adjacent segments [16]. With the emergence of minimally invasive spinal endoscopic techniques, new treatment options have become available for LDD.

Minimally invasive techniques such as UBE/BESS and OSE have been widely used in the treatment of lumbar disc disease (LDD) with good results [6, 17, 18]. AUSS, a new technique introduced in 2021, offers less invasiveness, a clearer surgical field, and higher procedural efficiency. Under the AO Spine nomenclature (Hofstetter et al., 2020), AUSS is best classified as an endoscope-assisted, single-portal technique-a category distinct from full-endoscopic working-channel procedures. We acknowledge that AUSS does not fit the strict definition of a working-channel full-endoscopic procedure per the AO Spine consensus. Rather, AUSS employs an arthroscope for visualization with instruments outside the scope, and is better characterized as an endoscope-assisted, single-portal technique (also termed uni-portal non-coaxial spinal endoscopic surgery, UNSES). Its comparison with UBE/BESS is justified by their shared minimally invasive nature and overlapping clinical indications. However, there is currently a lack of systematic evaluation of the efficacy and safety of AUSS in the treatment of LDD. This study is the first to use a meta-analysis to compare the efficacy and safety of AUSS and UBE/BESS in the treatment of lumbar degenerative disc disease, incorporating a total of 3 studies involving 810 patients.

These results indicate that both AUSS and UBE/BESS are safe and effective minimally invasive techniques for treating lumbar degenerative diseases. However, AUSS offers advantages in terms of surgical efficiency: its single-incision design eliminates the time required to establish dual access channels, reduces the need for intraoperative fluoroscopic guidance, causes less damage to paraspinal muscles, and results in less intraoperative bleeding. From a health economics perspective, reductions in operative time and intraoperative blood loss could potentially translate into resource savings. However, no formal cost-effectiveness or cost-utility analysis was performed in this review, and any claims of economic superiority would be speculative. Formal health economic evaluations are warranted to determine whether the observed efficiency advantages of AUSS confer actual cost benefits in different healthcare settings.

Regarding the 3-month low back pain VAS score, although the difference between groups reached statistical significance (MD = − 0.14, *P* = 0.01), the clinical relevance of this finding deserves careful consideration. The minimal clinically important difference (MCID) for VAS has been estimated to range from 1.5 to 2.0 points in patients with lumbar degenerative conditions, depending on the specific population and methodological approach used [19]. The observed difference of 0.14 points falls substantially below this range, suggesting that the statistical significance observed likely reflects the relatively large sample size (810 patients) rather than a clinically meaningful advantage in pain relief. This interpretation is further supported by the comparable ODI scores and leg pain VAS scores across all postoperative time points, as well as the comparable low back pain VAS scores at 6 months. Collectively, these findings indicate that while AUSS may offer certain perioperative efficiency benefits, it does not confer a clinically meaningful advantage in patient-reported pain outcomes compared with UBE/BESS.

The advantages of AUSS in terms of operative time, blood loss, and incision length can be explained from a technical design perspective. First, AUSS employs a single-incision design, which eliminates the time required to establish dual channels in UBE/BESS and the need for repeated fluoroscopic guidance during the procedure, thereby directly reducing the number of steps and the length of the incision [20, 21]. Second, AUSS incorporates the triangular operating concept of arthroscopy, allowing the surgeon to perform coordinated two-handed maneuvers—holding the scope in one hand and the instrument in the other—while switching roles at any time [8]. This flexibility, which is difficult to achieve with single-channel coaxial endoscopes, enables the surgeon to perform decompression procedures more efficiently. Third, AUSS provides a field of view closer to that of open surgery, reducing the difficulty of hand-eye coordination and helping to shorten the learning curve [22]. The reduction in CK and CRP levels observed in this study objectively confirms that AUSS involves less dissection and traction of the paraspinal muscles, a finding closely related to its single-incision design and the protection of muscle attachment sites [15, 23].

To date, no meta-analysis directly comparing AUSS and UBE/BESS has been published; however, existing meta-analyses comparing OSE and UBE/BESS provide some reference [10]. That study found that OSE was superior to UBE/BESS only in terms of incision length, while there were no significant differences in operative time or intraoperative blood loss. The results of this study are partially consistent with those findings, as AUSS also demonstrated an advantage in incision length. However, this study also found that AUSS resulted in shorter operative times and lower intraoperative blood loss compared to UBE/BESS, which may be related to technical details: although OSE and AUSS both belong to the category of single-incision, single-channel, non-coaxial endoscopic techniques, they differ in terms of operational integration. OSE employs a single-incision, dual-channel design, retaining two independent entry points within the same skin incision: the observation channel and the working channel run in parallel [20]; whereas AUSS further integrates both into a single flexible incision, achieving true single-channel operation. More importantly, AUSS incorporates a 30 arthroscope and unique non-coaxial rotation technology [8], allowing the surgeon to perform coordinated two-handed maneuvers—holding the scope in one hand and the instrument in the other—while being able to switch roles at any time, resulting in greater flexibility and efficiency. This difference in operational integration and flexibility may be one of the reasons why AUSS significantly outperforms UBE/BESS in terms of operative time, whereas OSE has not achieved such significant advantages.

In addition to the three direct comparison studies included in this meta-analysis, Zhou et al. also published a single-arm study on the treatment of lumbar disc herniation complicated by lateral recess stenosis using AUSS, which systematically analyzed occult blood loss following AUSS. The study included 129 AUSS patients and found that the average occult blood loss reached 414.34 mL, accounting for more than 93% of total blood loss [24], suggesting that although AUSS is a minimally invasive procedure, its occult blood loss should not be overlooked. The study also identified operative time and ASA classification as independent risk factors for occult blood loss. Although this study was single-arm in design and lacked a UBE/BESS control group, making direct comparisons of relative efficacy impossible, the objective data it provided on the characteristics of perioperative blood loss and influencing factors in AUSS can serve as indirect support for this meta-analysis and provide a reference for designing high-quality controlled studies in the future. Since the study design did not meet the inclusion criteria for this meta-analysis, it was not included in the quantitative synthesis.

The high heterogeneity observed for several outcomes warrants further discussion. Based on the available data and clinical knowledge, we attribute the substantial between-study variability primarily to three factors: First, disease type heterogeneity—the studies by Wang et al. and Zhou et al. enrolled patients with lumbar spinal stenosis (LSS), requiring laminectomy, ligamentum flavum resection, and lateral recess decompression, whereas Yang et al. enrolled patients with lumbar disc herniation (LDH), requiring only discectomy. These fundamentally different surgical scopes inevitably influence operative duration and blood loss. Second, surgeon experience and learning curve effects—as AUSS was introduced only in 2021, proficiency varies considerably across centers. The wide range of reported AUSS operative times for LSS patients (50.2 ± 8.9 min in Wang et al. versus 90.09 ± 8.73 min in Zhou et al.) suggests that surgeon experience substantially influences procedural efficiency. Third, sample size imbalance—Wang et al.‘s study contributed 475 of 810 patients (58.6%), exerting disproportionate influence on the pooled estimates. These factors collectively limit the interpretability of the pooled estimates. Importantly, the leave-one-out sensitivity analysis confirmed that the direction of the effect was consistent across all exclusion scenarios, suggesting that the observed AUSS advantages are not driven by any single study. However, the magnitude of the effect should be interpreted with caution, and the pooled estimates should be viewed as reflecting overall trends rather than precise effect sizes. It is also important to acknowledge that the three included studies encompassed different pathological subtypes-two focused on lumbar spinal stenosis (LSS) and one on lumbar disc herniation (LDH). These conditions differ fundamentally in surgical scope: LSS typically requires laminectomy, ligamentum flavum resection, and lateral recess decompression, whereas LDH often requires only discectomy. Pooling these heterogeneous populations may reduce the clinical interpretability of the pooled estimates. We considered performing subgroup analyses according to disease type; however, this was not feasible due to the limited number of included studies (only three studies, with only one focusing on LDH). Consequently, the pooled estimates should be interpreted as reflecting overall trends across a mixed patient population rather than disease-specific effects. Future studies with larger sample sizes should explore whether the observed advantages of AUSS are consistent across different pathologies.

The findings of this study provide evidence-based guidance for spinal surgeons in selecting surgical approaches. For patients and surgeons seeking less invasive procedures and shorter operative times, AUSS may be a preferable option to UBE/BESS. In particular, AUSS may demonstrate greater advantages in the multilevel decompression of lumbar spinal stenosis. However, the long-term efficacy of both techniques requires further validation. Furthermore, as AUSS is a new technique introduced only in 2021, its learning curve, long-term complications, and revision rates require confirmation through studies with larger sample sizes and longer follow-up periods.

It is also worth emphasizing that the observed advantages of AUSS are confined to surrogate surgical outcomes—operative time, blood loss, and incision length—which, while relevant to surgical efficiency, do not necessarily reflect superior patient-centered benefits. The comparable ODI, pain VAS, hospital stay, and complication rates between the two groups suggest that both techniques achieve similar clinical effectiveness from the patient’s perspective. Therefore, while AUSS may offer procedural efficiencies that are attractive to surgeons and healthcare systems, these should not be overinterpreted as evidence of clinically superior outcomes for patients. Future studies should prioritize patient-reported outcome measures and long-term functional results to determine whether the efficiency advantages of AUSS ultimately confer meaningful benefits to patients. We acknowledge that the high statistical heterogeneity (I² = 97–98%) observed for several outcomes limits the interpretability of the pooled estimates. However, meta-analysis remains valuable as it provides a quantitative summary of the available evidence, identifies patterns across studies, and generates hypotheses for future research. Given the early stage of evidence on AUSS, we present this as the first exploratory quantitative synthesis rather than a definitive comparative analysis.

### Limitations

All included studies were retrospective cohort designs; there were no randomized controlled trials, and selection bias and confounding factors may be present; the follow-up period was short (up to 12 months), making it impossible to assess long-term efficacy; All studies were conducted in China, limiting their generalizability; some outcomes exhibited extremely high heterogeneity, which could not be fully explained even after subgroup and sensitivity analyses; as fewer than 10 studies were included, publication bias could not be assessed, and publication bias may exist. We acknowledge that publication bias cannot be excluded. Although formal statistical tests were not performed due to the limited number of studies (*n* < 10), the risk of publication bias is heightened in this meta-analysis because AUSS is a novel technique developed in a single country, and early reports may preferentially publish positive experiences. Consequently, the pooled estimates-particularly for surgical efficiency outcomes-may be overestimated. Additionally, the inclusion of different pathological subtypes (LSS and LDH) within the same meta-analysis may limit the clinical interpretability of the pooled results, as these conditions differ in surgical complexity and expected outcomes. Subgroup analysis by disease type was considered but could not be performed due to the limited number of studies. This should be kept in mind when interpreting the findings. We acknowledge that formal GRADE assessment was not performed. Given the observational design of all included studies, the small number of studies, and the substantial heterogeneity, the overall certainty of evidence would likely be rated as low to very low by GRADE criteria. Therefore, our conclusions should be interpreted as exploratory, and high-quality prospective studies are needed to confirm these findings. Du Jin et al. reported clinical observations on the treatment of lumbar disc herniation using AUSS and UBE/BESS; however, the incision length in their UBE/BESS group was 6.02 ± 0.32 cm, far exceeding the incision length of conventional dual-channel endoscopic surgery. Furthermore, the standard deviation was only 0.32, indicating an overly concentrated data distribution that differed significantly from similar studies, suggesting potential data recording or measurement bias. Additionally, the article was published in a non-core journal, with a brief methodological description lacking key information such as follow-up duration and control of confounding factors. To ensure the robustness of the meta-analysis results, this study did not include it in the quantitative synthesis. Despite these limitations, this study remains the first meta-analysis comparing the clinical efficacy and complications of AUSS and UBE/BESS techniques, providing valuable reference information for clinical decision-making.

Based on the findings and limitations of this study, future research should focus on the following areas: First, conduct large-scale, multicenter, prospective randomized controlled trials to compare the long-term efficacy (≥ 2 years) of AUSS and UBE/BESS; second, standardize the reporting criteria for outcome measures, particularly the definition and grading of complications; third, focus on patient-reported outcomes, such as time to return to work and satisfaction; Fourth, compare the differences in efficacy between the two techniques in different patient subgroups (e.g., elderly, obese, or patients with multilevel disease); fifth, conduct health economic evaluations to compare the cost-effectiveness of the two techniques. Additionally, an ongoing interventional study (ChiCTR2500107269) aims to investigate the clinical outcomes of unilateral versus bilateral fixation in AUSS endoscopic fusion, and its results will provide high-level evidence for the further optimization of AUSS technology. Another ongoing observational study (ChiCTR2600116944) aims to compare neurophysiological monitoring during AUSS and UBE/BESS for the treatment of herniated discs; its results are expected to provide objective neurophysiological evidence regarding the impact of the two procedures on neurological function.

## Conclusion

In summary, this exploratory meta-analysis suggests that both AUSS and UBE/BESS are safe and effective minimally invasive options for lumbar degenerative diseases, with comparable functional recovery and safety profiles. AUSS appears to be associated with improved surgical efficiency metrics—shorter operative time, smaller incisions, and less intraoperative bleeding—in selected Chinese retrospective cohorts. However, these surrogate outcomes do not translate into superior patient-centered outcomes, as ODI, pain scores, hospital stay, and complication rates were comparable between groups. Given the limited number of included studies, the absence of RCTs, the single-country origin of all studies, and the substantial heterogeneity observed, the current evidence is of low certainty and does not support definitive conclusions of clinical superiority. Our findings should be considered hypothesis-generating, and large-scale, multicenter prospective studies are urgently needed to confirm whether the observed efficiency advantages of AUSS translate into meaningful patient benefit.

## Data Availability

The datasets generated and analysed during the current study are available from the corresponding author on reasonable request.
